# Mesenchymal stem cell therapy in the treatment of osteoarthritis: reparative pathways, safety and efficacy – a review

**DOI:** 10.1186/s12891-016-1085-9

**Published:** 2016-05-26

**Authors:** Julien Freitag, Dan Bates, Richard Boyd, Kiran Shah, Adele Barnard, Leesa Huguenin, Abi Tenen

**Affiliations:** Melbourne Stem Cell Centre, Level 2, 116-118 Thames St, Box Hill North, VIC 3128 Australia; Monash University, Melbourne, Australia; Magellan Stem Cells, Melbourne, Australia; Metrospinal Clinic, Melbourne, Australia

**Keywords:** Mesenchymal Stem Cells, Osteoarthritis, Knee

## Abstract

Osteoarthritis is a leading cause of pain and disability across the world. With an aging population its prevalence is likely to further increase. Current accepted medical treatment strategies are aimed at symptom control rather than disease modification. Surgical options including joint replacement are not without possible significant complications. A growing interest in the area of regenerative medicine, led by an improved understanding of the role of mesenchymal stem cells in tissue homeostasis and repair, has seen recent focused efforts to explore the potential of stem cell therapies in the active management of symptomatic osteoarthritis. Encouragingly, results of pre-clinical and clinical trials have provided initial evidence of efficacy and indicated safety in the therapeutic use of mesenchymal stem cell therapies for the treatment of knee osteoarthritis. This paper explores the pathogenesis of osteoarthritis and how mesenchymal stem cells may play a role in future management strategies of this disabling condition.

## Background

Osteoarthritis (OA) is a major cause of disability and chronic pain. With advances in modern medicine improving the prevention, diagnosis and treatment of many diseases that were once life-threatening, the population is now living longer. This increased life expectancy has led to an increased burden of degenerative conditions including osteoarthritis.

It is estimated that at least 27 million people across the United States of America are affected by arthritis, with an estimated total annual cost to the US economy of $89.1 billion US dollars [1]. Worldwide, arthritis is considered to be the fourth leading cause of disability [2]. In both the developed and developing world, osteoarthritis is an important factor affecting disability-adjusted life years [3].

Osteoarthritis is a progressive and painful condition that can affect both the young and the old and is a highly prevalent condition in the Western world. It has a radiological prevalence of up to 80 % in subjects over the age of 65 years [4–6]. Symptomatic osteoarthritis affects 10 % of males and 18 % of females over the age of 45 years [7]. Prevalence is likely to further increase given the increasing proportion of older people in society [4, 5].

Current medical treatment strategies for OA are aimed at pain reduction and symptom control rather than disease modification. These pharmaceutical treatments are limited and can have unwanted side effects [8, 9]. Viscosupplement/hyaluronic acid (HA) intra-articular injections have been used to treat symptoms of mild to moderate knee OA, however, their mechanism of action is uncertain, with some studies suggesting little improvement beyond that achieved with placebo injections [10].

Methods used for repair of articular cartilage lesions include autologous chondrocyte transplantation, microfracture, and mosaicplasty. These techniques are, however, limited to the repair of focal defects and consequently we lack a reparative technique for the more global/diffuse pathology of OA.

Surgical total knee replacement (TKR) is the current accepted treatment of choice for symptomatic knee OA that is not controlled by traditional conservative therapies. It is estimated that approximately 600,000 TKR procedures are performed annually in the US [11]. Alarmingly – and perhaps reflecting increased rates of obesity - an increasing proportion of patients who undergo a TKR are under the age of 65 [12]. Further, revision rates of primary TKR are 2.5 times higher in patients under 65 years of age [13]. Not surprisingly it is estimated that the number of annual total knee revision operations performed will grow by over 600 % between the years 2005 and 2030 [14].

Total knee replacements are not without significant complication [15, 16]. As many as 20 % of patients will continue to have knee pain and other problems post TKR [17]. Significant complications such as death, pulmonary embolism and infections requiring readmission to hospital occur in up to 2 % of patients [18].

The health and economical impact of OA has seen it become an international public health priority and has led to the active exploration and research of alternative regenerative and joint preservation therapies including mesenchymal stem cells.

## Pathobiology of osteoarthritis

Osteoarthritis is characterized by progressive and irreversible cartilage degeneration. The capacity of articular cartilage to repair is inherently poor, with the relative avascularity of cartilage, and hence lack of systemic regulation, likely leading to an ineffective healing and reparative response [19, 20].

Structurally the changes of OA are observed as combinations of the following: loss of cartilage thickness, peri-articular bone formation (osteophytes), subchondral sclerosis, cyst formation and peri-articular tissue changes (i.e., synovitis) [21].

Whilst both mechanical, genetic and other factors influence development of OA, the primary risk factor is age [22]. Components of the cartilage extracellular matrix (ECM) including type II collagen and proteoglycans undergo age related structural changes, leading to likely alteration in the biomechanical properties of the ECM [23]. Advanced glycosylation end products also accumulate within cartilage, leading to increased cross-linking and altered biomechanical properties [24]. These changes lead to a loss in the ability of cartilage to adapt to mechanical stress/load.

Chondrocytes within the cartilage matrix also exhibit age related changes. It has been proposed that reactive oxygen species (free radicals) induced by mechanical or biological stressors may lead to cell senescence [25]. Cell senescence is accompanied by reduced growth factor response and production, coupled with an observed upregulation of inflammatory cytokine expression such as Interleukin-1 (IL-1), Tumor Necrosis Factor Alpha (TNFα) and Matrix Metallopeptidase -13 (MMP-13) [26, 27]. IL-1 and TNFα are primary drivers of a cytokine led degradation of cartilage [28].

These cytokines also directly stimulate the production of other pro-inflammatory factors including IL-8, IL-6, leukotriene inhibiting factor, proteases and prostaglandin E2 (PGE2). IL-1 and TNFα both increase synthesis of MMP and decrease MMP enzyme inhibitors, resulting in a net catabolic environment and loss of extracellular matrix [28]. MMP-13 serves as a major mediator of type II collagen cleavage and matrix degradation [26, 29]. Another catabolic cytokine MMP-7 (mattrolysin) has been localized to chondrocytes in the superficial and transitional layers in OA but not the deeper layers [30].

Nitric Oxide (NO) is a free radical that has also been implicated in the pathology of OA. Both NO and NO Synthase are synthesized by chondrocytes. NO has an ability to inhibit proteoglycan synthesis and also to inhibit the effect of IGF-1 on chondrocytes. It is thought to also perhaps play a role in the apoptosis of chondrocytes [31, 32]. Further, chondrocyte apoptosis leads to the formation of apoptotic bodies which express catabolic properties. These may contribute to the observed abnormal chondral calcification and osteophyte formation that is seen in OA [32].

Evidently there are a host of enzymatic compounds that are involved in the disruption of the collagen matrix leading to the degradative process of OA. However, despite OA being considered a degenerative condition, several studies have confirmed that in areas of OA, many chondral cells demonstrate enhanced synthesis of extracellular matrix components [33–39]. This anabolic response, however, seems to be limited to the deeper chondral zones, with the upper zones exhibiting reduced expression of matrix components such as agrecan [28, 40].

Whilst chondrocytes may remain active in the area of OA, research has indicated that they can undergo dedifferentiation as a result of interaction with the changing ECM environment. Chondrocytes in the upper to middle zones are seen to express type III rather than type II collagen and in fact those cells in the deeper zones display Type X collagen expression - typical of cartilage within growth plates and prone to ossification [28, 41].

These observed differences in anabolic and catabolic processes, and presence of degradative cytokines within chondrocytes of differing layers, may explain the progressive nature of OA from superficial to deep zones.

Changes of osteoarthritis are not only limited and influenced by the cartilage environment. It is understood that the process of degeneration is also under the influence by the release of pro-inflammatory mediators from the synovium. This seems in part the effect of synovial originating cytotoxic M1 macrophages on the down-regulation of chondrogenic gene expression of mesenchymal stem cells (MSCs) [42]. Low-grade synovial inflammation – observed in OA - is also associated with increased expression of catabolic mediators including PGE2, NO and neuropeptides [43].

Interestingly, evidence indicates that osteoarthritis is associated with a depleted local population of stromal MSCs, and those that exist exhibit reduced proliferative and differentiation capacity [44, 45]. The depletion and functional alteration/down regulation of MSC populations with reduced differentiation capacity has also been postulated as a cause for progressive degenerative OA [46, 47]. Despite these findings, it has been noted that there exists MSCs with chondrogenic differentiation potential in patients with OA, irrespective of age or the etiology of disease [48].

Other important contributing factors which affect both the onset and progression of OA – but which are not a focus of this article - include obesity, history of trauma, genetics, muscle weakness and various heritable and acquired disorders [49].

Simplistically it is accepted that OA occurs when there exists an imbalance between inflammatory/catabolic and anabolic pathways. Age related loss of the ability of chondrocytes and tissues within the ECM to maintain a homeostasis between these pathways, leads to a pro-catabolic state favoring matrix degradation [50]. This loss of homeostasis and inability to adapt to external mechanical stressors results in the development of OA.

Acknowledgement of this imbalance between catabolic and anabolic pathways has led to renewed interest in therapies that may be able to influence and encourage maintenance of an appropriate chondral homeostasis.

## Mesenchymal stem cells

### Mesenchymal stem cell properties

Regenerative cellular therapies, rather than being unique and experimental, are well established and practiced in the area of blood transfusion, bone marrow and tissue transplantation and reproductive in-vitro fertilization.

It has been over 40 years since mesenchymal stem cells were first characterized by Dr Alexander Friedenstein. They were initially recognized in bone marrow and display plasticity and multipotency. Similar cells have been shown to be present in other tissues including peripheral blood, cord blood, skeletal muscle, heart and adipose tissue [51, 52]. The presence of these cells within other tissues has meant that they are perhaps more accurately described as mesenchymal stromal cells.

MSCs are able to form cells of the mesodermal lineage, being able to differentiate towards osteoblasts, chondrocytes and adipocytes [52–54]. Their presence throughout the body suggests an intrinsic role in tissue repair and regeneration.

Several in vitro techniques have been explored to assist MSCs to differentiate along a path of chondrogenesis. Both Transforming Growth Factor Beta 1 (TGFβ1) and Insulin-Like Growth Factor 1 (IGF-1) act synergistically to stimulate chondrogenesis. This is in part mediated by MAPKinase and Wnt signaling pathways [55, 56]. Importantly the expression of collagen type II and proteoglycans associated with hyaline cartilage are similar in in-vitro MSC derived chondrocytes to mature adult chondrocytes [56]. Other compounds found to assist in the propagation of MSCs along a chondrogenic lineage are dexamethasone [57], some bone morphogenic proteins (BMP) – primarily BMP-7 [58], and fibroblast growth factor (FGF-2) [59].

Whilst evidence of the capacity of MSCs to differentiate along a chosen cell lineage represents great promise in the area of regenerative medicine it is postulated that their beneficial effect is also achieved through an immunomodulatory and paracrine mechanism and hence manipulation of the disease process [60].

MSCs are observed to suppress inflammatory T–cell proliferation, and inhibit maturation of monocytes and myeloid dendritic cells resulting in an immunomodulatory and anti-inflammatory effect. This immunomodulatory mechanism raises potential for their use in auto-immune mediated inflammatory conditions including inflammatory arthropathies [61].

Along with their immunomodulatory and differentiation potential, MSCs have been shown to express essential cytokines including Transforming Growth Factor beta (TGFβ), Vascular Endothelial Growth Factor (VEGF), Epidermal Growth Factor (EGF) and an array of bioactive molecules that stimulate local tissue repair [62–64]. These trophic factors, and the direct cell to cell contact between MSCs and chondrocytes, have been observed to influence chondrogenic differentiation and cartilage matrix formation [65, 66]. Importantly, analysis of mRNA levels within cartilage chondrocytes present at end stage arthritis, indicates that endogenous cells are not inert and remain metabolically active and continue to synthesize cartilage proteins. This supports the hypothesis that MSCs may be able to assist the existing chondrocytes - much like what is observed in their perivascular stromal role within the bone marrow.

Indeed, the anti-inflammatory, anti-apoptotic, and anti-fibrotic mechanisms influenced by the properties of MSCs may be their primary mode of activity [67].

Autologous MSCs can differentiate into cartilage and bone supporting their potential in the treatment in OA [68, 69]. Further research highlighting the pro-inflammatory cytokines involved in the destruction of hyaline cartilage and development of degenerative osteoarthritis has also highlighted the potential of MSCs as a disease modifying agent due to their immunomodulatory/anti-inflammatory properties [27]. An ability to migrate to sites of injury, inhibit pro-inflammatory pathways and promote tissue repair through release of anabolic cytokines and direct differentiation into an array of specialized connective tissue cells, has led to renewed focus on MSCs in the area of regenerative medicine.

### Mesenchymal stem cell characterization

MSCs are a heterogeneous population of cells that lack a specific and unique marker. It is postulated that it is their heterogeneity that allows MSCs to respond to a wide variety of cues in the local environment, and therefore carry out a number of functions [70].

MSCs are characterized by their plastic adherent properties and expression of several surface antigens including CD105, CD 90 and CD73, and their absence of hematopoietic markers CD34, CD45, CD14 or CD11b, CD79α or CD19 and also the absence of HLA Class II molecules [71].

The international Society of Cellular Therapy has proposed that the MSC population must exhibit at least ≥95 % expression of CD105, CD73 and CD 90 and ≤2 % of hematopoietic markers for an accepted level of purity. Further, these cells must be able to show an ability to differentiate along osteogenic, chondrogenic or adipogenic cell lines [71].

### Source of mesenchymal stem cells

Mesenchymal stem cells are found throughout the adult body – hence they are often referred to as mesenchymal stromal cells. The ability to use adult MSCs placates the ethical concerns of using embryonic stem cells. The best source of adult MSCs, however, remains unclear. Several different tissues have been explored including bone marrow, adipose tissue, and umbilical cord tissue (Wharton’s jelly).

Traditionally bone marrow has been used as a source of MSCs, though research has shown a relative paucity of MSCs within bone marrow aspirates (BMA) – comprising only .001–.02 % of mononucleated cells isolated from aspirates [72, 73]. In comparison, human adipose tissue through a lipoaspirate procedure, yields MSC numbers of ~ 1–7 % of the nucleated cell population [74]. Its ease of harvest and the relative abundance of MSCs in adipose tissue has seen this method increasingly used for autologous therapies.

Whilst past research has indicated bone marrow MSCs to have superior chondro-progenitor capacity, a number of recent publications have indicated comparative chondrogenic ability of MSCs from either bone marrow or adipose tissue [48, 74–77].

Past research has indicated that MSCs exhibit reduced proliferative and differentiation capacity with age [44, 45] – with some authors proposing this as a cause of age related degenerative conditions. Human umbilical cord perivascular cells (HUCPVCs) – otherwise known as Wharton’s Jelly – are a rich source of mesenchymal stem cells [78]. HUCPVCs are closer to an embryonic cell lineage and are robust/stable, show increased differentiation capacity and retain properties of true stem cells even after extended in-vitro expansion/culture [79]. Further, HUCPVCs appear to lack tumorgenicity and, even when used in the presence of cancer, are not associated with enhanced growth of solid tumors [80].

Like MSCs of other origins, HUPVCs are hypo-immunogenic and therefore offer promise as an allogeneic source. MSCs are negative for HLA Class II surface antigens and express only low levels of HLA Class I antigens [81]. Perhaps surprisingly, as MSCs differentiate towards chondrocytes, adipocytes or osteocytes, they continue to be non-immunogenic and lack HLA Class II expression.

The chosen source of MSCs is dependent upon ease of harvest and the differentiation capacity towards a chosen tissue. Whilst autologous therapies offer an attractive option, the cost of individual harvest, isolation and expansion of cells in an appropriate `clean facility’, is obstructive. Allogeneic MSC therapies  may offer accessibility of disease modifying regenerative therapies to the broader community.

## Current regenerative techniques

With an aging population, and an alarmingly increasing rate of total joint replacements being performed on those under the age of 65, there has been significant focus on regenerative joint preservation techniques. These include: autologous chondrocyte transplantation (ACT), mosaicplasty, and microfracture. Whilst they are limited to isolated areas of chondral loss and are less adaptable to the generalized degenerative changes as seen in arthritis they are often considered, when clinically appropriate, in an attempt to improve both pain and function, delay progression to arthritis and therefore to delay the later need for total joint replacement. Whilst not a focus of this review, as current mesenchymal stem cell based therapies are often modeled and compared to these techniques, it is important to understand the theory and observed clinical efficacy of these accepted surgical approaches.

### Autologous chondrocyte transplantation

ACT involves the autologous harvesting of cartilage from a non-weight bearing area. Chondrocytes are then isolated from the cartilage and seeded in vitro in monolayer culture and expanded. They are injected into the chondral defect and a cover – traditionally a periosteal flap – is then sutured in place to secure the chondrocyte graft [82].

Preclinical trials have successfully shown this method to be successful in resulting in hyaline like cartilage regrowth/repair compared to control groups [83–85]. ACT clinical results have correspondingly been encouraging with reasonable observed long-term durability [82, 86]. However, despite these encouraging clinical outcomes, there remains a lack of comparative, controlled, long-term clinical studies.

ACT is limited by the paucity of autograft donor sites, damage caused by the technique of harvesting and at times poor integration of the grafted defect with surrounding cartilage [87]. Further, studies have indicated that up to 40 % of ACTs show evidence of chondrocyte dedifferentiation. This may be linked to the down regulation of chondrocytes during ex vivo culture resulting in the production of collagen type I rather than type II [88, 89]. This down regulation of chondrocytes is not only an effect of dedifferentiation during the monolayer expansion phase but is also understood to be due to the loss of interaction between the implanted chondrocyte and a normal surrounding ECM.

Down regulation of chondrocytes with expression of type I collagen may lead to formation of fibrocartilage rather than hyaline cartilage, with resultant reduced load bearing properties. Roberts and colleagues showed varying histology of ACT sites biopsied up to 34 months post implantation with predominantly hyaline features in 22 % of specimens, fibrocartilage formation in 30 % and a mixed collagen population in 48 % of samples [90].

Donor site morbidity, down regulation of chondrocytes with fibrocartilage formation and poor integration has meant that we continue to need to explore and development other alternative techniques in chondral defect repair. A further limitation of ACT is that its current use in the treatment of isolated chondral defects does not easily translate to treatment of the more global chondral degenerative changes as found in generalized OA.

### Microfracture

Microfracture – otherwise known as osteoplasty - has become a commonly used surgical technique to assist in stimulating a healing response at the site of an isolated chondral defect. The procedure involves the drilling or punching of holes through the subchondral plate at the site of a full thickness chondral defect. This stimulates an inflammatory response, and the subsequent migration of bone marrow derived pluripotent cells to the articular surface creates an environment amenable to healing [91].

Whilst several studies have successfully demonstrated a cartilaginous response at the sites of microfracture, histological analysis has suggested that the resultant tissue is consistent with collagen type I fibrocartilage rather than the hyaline – collagen type II - cartilage typical of normal articular surfaces [92, 93]. Although effective short to medium term functional improvement of joint function has been noted following microfracture, long-term results are less encouraging. Follow-up of 33 ankles post arthroscopic microfracture for ankle talus lesions found a disappointing fair to poor clinical outcome in 54 % of patients at a mean follow up of 66 months [94].

Inadequate defect filling, and the poor load bearing quality of fibrocartilage with early degeneration, have been postulated as reasons for poor long-term outcome following microfracture [95, 96].

### Mosaicplasty

Mosaicplasty involves the use of autologous osteochondral grafts to an area of full thickness chondral loss of up to 9 mm. Grafts are taken from areas of non-weight bearing at the periphery of the joint and transplanted to the site of the defect. It is expected that fibrocartilaginous growth will occur between these grafts, acting as `grouting’ for the mosaicplasty [97].

Several follow up studies have, however, indicated the resorption of the chondral layer of the graft and degeneration of the surrounding chondral surface [98, 99]. A randomized controlled trial comparing mosaicplasty versus ACT in osteochondral defects of the knee, demonstrated at 12 months follow-up arthroscopy excellent or good results in 82 % of patients who received ACT versus only 34 % patients after mosaicplasty [100]. As ACT techniques have also shown success even in areas of osteochondral loss with significant depth of cancelous defect, the reasoning to perform mosaicplasty is less apparent.

## MSCs and cartilage repair

MSCs, due to ease of harvest and isolation with minimal donor site morbidity, coupled with an ability to expand into chondrocytes, have meant that they have been actively explored in regards to tissue engineering and repair.

### MSC scaffold transplantation techniques – preclinical results

Preclinical trials using techniques similar to ACT, but substituting the chondrocytes with MSCs, have shown positive results with formation of tissue with histological properties consistent with hyaline cartilage and a high type II collagen presence [101, 102]. The efficacy of mesenchymal cellular scaffold constructs has been further substantiated with a porcine model, which again showed hyaline like cartilage regeneration at 3 and 6 months post implantation [103].

Dragoo and colleagues used isolated and expanded adipose derived MSCs in fibrin glue to treat chondral defects in rabbits [104]. Post treatment histological analysis showed hyaline like cartilage repair in 12 of 12 subjects, versus only 1 in 12 control subjects, supporting the use of cellular tissue matrixes in tissue engineering. Other studies, which have pre-differentiated the MSCs towards chondrocytes prior to implantation, have similarly shown success [105–107].

### MSC scaffold transplantation techniques – clinical results

The results of initial clinical studies have reflected the results of preclinical trials. Wakitani and colleagues successfully transplanted isolated MSCs - seeded onto a type I collagen network - to an area of chondral defect, resulting in successful filling of the defect [108]. Later biopsy at two years indicated hyaline like cartilage with type II collagen on histological evaluation.

Nejadnkik and colleagues published their results of a comparative cohort study assessing both the safety and efficacy of bone marrow MSC impregnated scaffolds (*n* = 36) in direct comparison to autologous chondrocyte transplantation (*n* = 36) for an isolated chondral defect [109]. There was no difference between these groups in clinical outcome.

Interestingly, these positive findings, however, are in contrast to earlier research that suggested transplanted MSCs might result in hypertrophic chondrocyte differentiation and expression of collagen type X [110]. Collagen Type X is associated with endochondral ossification [111].

### MSC injectable techniques – preclinical results

Recognizing the limitation of biological scaffolds in the treatment of OA – where there exists more diffuse cartilage loss rather than an isolated cartilage lesion - other researchers have sought to assess the effect of intra-articular MSC injections.

Preclinical trials have successfully indicated the benefit of MSC intra-articular injections on improvement in function, though results have been inconsistent on cartilage restoration. Some studies, whilst indicating significant pain and functional improvement, have not seen any observable difference in disease progression against controls, whilst others have successfully shown disease modification.

In a mono-iodoacetate induced rat model of OA, use of intra-articular bone marrow MSCs, resulted in animals being able to distribute significantly greater weight through the affected limb. In contrast to this functional improvement, no statistically significant difference between the treatment and control groups, in regard to cartilage and subchondral bone pathology and synovial inflammation, was observed [112].

In a surgically induced model of OA in the goat, intra-articular injections of labeled bone marrow MSCs resulted in regeneration of chondral tissue in comparison to the control group. This observation was made despite the relative lack of labeled MSCs being later found within the regenerative cartilage area [113]. Further, in a later porcine model, MSC injectable therapies again showed preclinical efficacy with improved cartilage healing of chondral defects when compared to control [114].

The use of MSC based therapy in conjunction with the accepted surgical technique of microfracture has been explored in a surgically induced isolated chondral lesion goat model. Post microfracture intra-articular injections of bone marrow aspirate (BMA) in combination with hyaluronic acid resulted in both improved integration of tissue and superior quality of tissue repair with type II collagen represented on histology [115].

Black and colleagues assessed the clinical effect of adipose derived MSCs within a randomized, placebo controlled trial showing a significant improvement in lameness and range of motion in dogs following a single intra-articular adipose derived MSC injection [116].

### MSC injectable techniques – clinical results

Similarly to preclinical results, clinical trials using injectable MSC techniques have reproducibly shown pain and function improvements, though observation of disease modification has been less consistent.

Using a combination of both isolated bone marrow MSCs, BMA and platelet lysate, Centeno and colleagues have published the observed improvement in both chondral volume and meniscus volume in two limited case studies [117, 118]. In 2011, Centeno later published a case series of 339 patients, reporting that of those patients requiring total knee replacement (69 % of the patient cohort) only 6.9 % still required replacement surgery after MSC therapy. Sixty percent of patients reported >50 % pain relief and 40 % reported >75 % pain relief at 11 months [119].

The success of such combination therapy has also been indicated by a limited case series assessing the benefits of adipose derived MSC, where MSC was combined with both a platelet lysate and a hyaluronic acid carrier with additional use of low dose dexamethasone [120]. Again, both functional and disease modification was observed.

Indication of disease modification has had further substantiation with Kuroda and colleagues successfully treating a femoral condyle cartilage defect with autologous bone marrow MSCs, showing repair with `hyaline-like’ tissue at later arthroscopy and biopsy [121]. In another study, use of a single intra-articular injection of autologous isolated expanded bone marrow derived MSCs resulted in both pain and functional improvement in all patients and increased cartilage thickness in 3 out of 6 patients [122]. The authors of this article, however, did note an increase in pain after 6 months, suggesting that a repeat injection may be of benefit.

Extending upon the observed positive preclinical outcome of the use of MSCs in conjunction with arthroscopic techniques, Saw and colleagues have recently published a randomized controlled trial involving the use of peripheral blood MSCs in combination with arthroscopic microfracture/microdrilling of chondral lesions. Importantly, the participant group receiving MSCs showed significant improvement in the quality of articular cartilage repair (by histological and MRI evaluation) in comparison to the control group that underwent microfracture and hyaluronic acid injections alone [123].

A randomized clinical trial assessing the efficacy of MSCs post arthroscopic partial medical meniscectomy, showed improvement in clinical outcome in comparison to control but also evidence of regeneration of meniscal volume [124].

Most recently, Phase I and II trials using expanded adipose derived MSCs in the treatment of OA have shown MRI evidence of cartilage regrowth [125]. Following a single intra-articular injection of 100 million MSCs, radiological (MRI) follow-up at 6 months showed increased cartilage volume and histological assessment confirmed hyaline–like cartilage regeneration with the presence of type II collagen.

Similarly, the use of allogeneic bone marrow MSCs in symptomatic osteoarthritis that was unresponsive to conservative management, has resulted in both pain ad functional improvement and significant improvements in cartilage quality on T2 MRI cartilage mapping at 12 months in comparison to controls [126].

These positive results showing disease modification are in contrast to a limited case series of four patients, where each patient received isolated adipose derived MSCs. Whilst functional improvement was noted at follow up, no structural change and joint space improvement was noted at repeat imaging – though this only involved X-ray rather than MRI [127]. The authors acknowledged that cell number, use of co-stimulators/carrier media (i.e., Platelet Lysate), the number and frequency of injections, and also stage of disease, might have influenced outcome.

A recent Phase 1 dosing trial on the use of adipose derived MSCs in severe osteoarthritis indicated a significant effect over a 12 month follow-up on the need for total joint replacement with only 2 out of the 18 patients still requiring arthroplasty [128]. This is similar to Centeno’s observation of the effect of MSC based therapy in delaying need for joint replacement.

Despite MSCs being commonly associated with regenerative medicine, and level IV evidence of chondral regrowth and disease modification, there is a paucity of well-controlled trials assessing structural outcome (see Table 1). Tucker and colleagues have appropriately highlighted that future research in the area of cellular therapies needs to focus on what they have termed an `outcome triad’ [129]. This includes - a) molecular and cellular responses both intra-articularly and systemically; b) clinical outcome – pain and function; c) structural outcome.

The reproducible pain and functional improvement seen with MSC injectable therapies, raises the question of whether the biological mechanism of action may be a strong anti-inflammatory effect - including on neurogenic inflammation – rather than regeneration. Further, the observed disease modification in studies that use combination therapy suggests that the efficacy of MSC therapies may be influenced by additional agents including platelet concentrates and hyaluronic acid - though this creates a further layer of confusion regarding cause and effect.

**Table 1:** Summary of regenerative techniques

Technique	Indication	Outcome	Level of evidence	Ref
Autologous Chondrocyte Transplantation	Isolated chondral defects	*Benefits* - can result in hyaline like cartilage formation - observed pain and functional improvement *Limitations* - donor site morbidity - poor integration with surrounding tissue - may result in fibrocartilage formation	Level II-V	[82–90]
MIcrofracture	Isolated chondral defects	*Benefits* - single stage surgical technique - observed pain and functional improvement *Limitations* - fibrocartilage formation - inadequate defect filling - poor long term outcome	Level I-V	[92–96]
Mosaicplasty	Isolated osteochondral defects	*Benefits* - use in deep osteochondral defects *Limitations* - graft resorption - donor site morbidity - poor long term outcome	Level II-V	[98–100]
MSC Scaffold Transplantation	Isolated chondral defects	*Benefits* - hyaline like cartilage repair - nil donor site morbidity - observed pain and functional improvement *Limitations* - potential chondrocyte hypertrophy	Level II- V	[108–111]
MSC Injectable Techniques	Isolated chondral defects Osteoarthritis	*Benefits* - use in generalized arthritis - relatively simple application - observed pain and functional improvement *Limitations* - limited evidence of efficacy - inconsistent observation of disease modification	Level II-V	[117–128]

## MSC + carrier media

### Platelet concentrate/platelet-rich plasma

The function of MSCs has been explored under the influence of bioactive carriers such as platelet-rich plasma (PRP). Platelets contain greater than 1500 protein based factors with bioactive ability [130]. This broad spectrum of compounds includes growth factors, peptide hormones, chemokines, fibrin and also proteins with anti-bacterial and fungicidal properties.

Growth factors released by platelets may potentially play a positive role in the up regulation of MSCs. TGFβ1 is seen to reduce collagen type I gene expression and up regulate expression of collage type II and aggrecan genes [131]. Further, TGFβ1 works in association with basic Fibroblast Growth Factor (FGF2) to assist in the migration of stromal cells to a site of injury [132, 133].

Importantly, whilst in vitro studies indicate the potential benefits of PRP in the modification of OA pathways, these preclinical results have not been observed in clinical trials where, despite an observed pain and functional improvement, PRP therapy in isolation has not been associated with disease modification and structural change.

The combination of PRP with MSCs in intra-articular injections has shown increased collagen type II expression and reduced chondrocyte apoptosis [134]. FGF2 also plays a critical role in suppressing collagen Type X formation and hence may also have an ability to prevent hypertrophic endo ossification [135]. Both symptomatic and structural improvement has been noted in a recent case series using a combination of PRP with MSC [136].

MSCs seeded in a PRP scaffold have been shown to both proliferate and express cartilage marker genes, resulting in improved cartilage differentiation and successful repair of chondral defects in rabbits [137]. Similar results were observed in an early pilot case study by Haleem and colleagues [138].

Further studies have indicated the combined benefits of using PRP in an ACT approach with a hydrogel scaffold seeded with both chondrocytes and PRP [139]. This application was used successfully in a broad cohort study of 81 patients with OCD of the ankle [140].

PRP has an anabolic effect on both chondrocytes and MSCs – assisting in proliferation, inhibiting deregulation and also assisting in matrix development that further supports appropriate chondrocyte and stem cell development.

The issue of PRP remains the variability in both its preparation and the resultant amount of bioactive factors that it expresses. Platelet count can also vary depending on the donor’s age, health, hydration and gender. Further, there are factors within PRP that may have unwanted effects on both the joints and MSCs – i.e. Vascular Endothelial Growth Factor.

### Hyaluronic acid

Preclinical studies have often used MSCs suspended in a hyaluronic acid (HA) based media with good efficacy. Murphy and colleagues showed successful regeneration of chondral tissue in a goat model with surgically induced OA [113]. Many clinical trials of MSC therapies have similarly used HA as a carrier media [120, 123].

The benefits of hyaluronic acid may be more than just its action as a carrier. Preclinical studies have observed both enhancement of synovial cell migration and chondrocyte migration with the application of HA in combination with FGF2 [141]. The observed interaction of HA with both MSCs and chondrocytes, through cell surface receptors CD44 and RHAMM (Receptor for Hyaluronic Acid Mediated Migration), indicates that HA may facilitate migration and adherence of MSCs to a chondral defect [114, 142–144].

Further, hyaluronic acid hydrogels have been shown to be an effective 3-dimensional environment in which MSCs both proliferate and express early changes associated with chondrogenesis [145].

## Safety

The investigation of MSCs in the treatment of various conditions including OA continues to grow. The National Institutes of Health lists 404 current trials in the area of MSCs [146]. With such continued interest in the possible clinical applications of MSC therapies, it is imperative to determine not just efficacy but also safety.

Rubio and colleagues in a controversial study in 2005 questioned the safety of adipose derived MSCs [147]. After in vitro culture over 4 months they demonstrated spontaneous stem cell transformation and development of malignancy when implanted in immune-deficient mice. Later this study was retracted after evidence indicated that the malignant transformation related to a contaminant cell line and not the MSCs [148]. In similar circumstances, a later study on long term cultured Bone Marrow MSCs - with evidence of malignant transformation - was retracted on identical grounds [149, 150].

A recent publication studying bone marrow and hepatic MSCs showed evidence of abnormal cell growth after culture beyond 5 weeks, with development of malignancy in immune-deficient mice [151]. They noted loss of MSC markers and also identified RNA/DNA gene sequences that may serve as biomarkers of cell transformation. In contrast to these findings, Bernado and colleagues demonstrated no abnormal growth of bone marrow MSCs after 25 passages or senescence and further culture for 8–12 weeks [152].

Importantly, based upon current clinical trial outcomes, MSC therapy appears safe. A recent systematic review and meta-analysis of trials involving a total of 1012 participants receiving intra-vascular MSC therapy for various clinical conditions including ischaemic stroke, Crohn’s disease, cardiomyopathy, ischaemic heart disease and graft versus host disease, did not identify any significant adverse events other than transient fever [153]. Patients were followed up in some studies for over 90 months. This meta-analysis included both autologous and allogeneic MSCs and also expanded/cultured cells.

Further, systematic review of clinical studies involving the use of intra-articular injections of autologous expanded MSCs, with a mean follow-up of 21 months of 844 procedures, showed no association with adverse events such as infection, death or malignancy [154].

Additionally, the use of carrier media’s such as PRP may improve safety further with PRP displaying both anti-bacterial and fungicidal properties [155].

## Conclusion

Osteoarthritis is a progressive and degenerative condition. With an aging population it promises to remain a significant cause of pain and disability. Whilst osteoarthritis is an active, inflammatory and progressive condition, there has been no development of disease modifying pharmaceutical therapies. Indeed, all currently accepted therapies are aimed at symptom control rather than disease prevention. Current conservative management strategies fail to alter disease progression and surgical management in the form of joint replacement is associated with not insignificant complications.

Methods for the repair of articular cartilage lesions – including surgical microfracture and cellular scaffold transplantation – have been investigated with success in both preclinical and clinical trials. Unfortunately, these techniques are limited to the repair of focal lesions only and are not easily transferable to osteoarthritis, where there is more generalized loss of cartilage volume.

Intra-articular injections of MSCs have resulted in pain and functional improvement in a number of preclinical and clinical trials. Importantly, recent limited case series evidence has shown regrowth of cartilage volume and disease modification following MSC injections. Whilst recognizing the low level of scientific evidence (Level IV), a significant increase in cartilage volume in an accepted degenerative and progressive condition represents an exciting development.

Despite initial concerns regarding MSC therapies, systematic review of clinical trials has indicated a relative safety in both intravascular and intra-articular injections. Evidence does support however that caution needs to be undertaken when culturing/expanding these cells.

The burden of musculoskeletal disease is progressively expanding and highlights the need for both preventative and reparative therapies rather than commonly accepted pain management interventions. MSC based cell therapies offer an exciting possibility in the treatment of OA and importantly show promise in disease modification, with potential inhibition of progression and recent evidence of reversal of this degenerative process. Importantly further randomized controlled trials are needed to evaluate the most effective application of MSCs in osteoarthritis management.

## Abbreviations

ACT, autologous chondrocyte transplant; BMA, bone marrow aspirate; BMP, bone morphogenic protein; ECM, extracellular matrix; EGF, endothelial growth factor; FGF2, basic fibroblast growth factor; HA, hyaluronic acid; HUCPVC, human umbilical cord perivascular cells; IL, interleukin; ILGF, insulin like growth factor; MMP, matrix metallopeptidase; MSC, mesenchymal stem cell; NO, nitric oxide; OA, osteoarthritis; PRP, platelet rich plasma; RHAMM, Receptor for Hyaluronic Acid Mediated Migration; TGF, transforming growth factor; TKR, total knee replacement; TNF, tumor necrosis factor; VEGF, vascular endothelial growth factor.
